# The widely distributed soft coral *Xenia umbellata* exhibits high resistance against phosphate enrichment and temperature increase

**DOI:** 10.1038/s41598-022-26325-5

**Published:** 2022-12-22

**Authors:** Selma D. Mezger, Annabell Klinke, Arjen Tilstra, Yusuf C. El-Khaled, Bianca Thobor, Christian Wild

**Affiliations:** 1 Department of Marine Ecology, Faculty of Biology and Chemistry, University of Bremen, Leobener Str. 6, 28359 Bremen, Germany; 2 Leibniz Centre for Tropical Marine Research, Fahrenheitstraße 6, 28359 Bremen, Germany

**Keywords:** Biogeochemistry, Ecophysiology, Metabolism

## Abstract

Both global and local factors affect coral reefs worldwide, sometimes simultaneously. An interplay of these factors can lead to phase shifts from hard coral dominance to algae or other invertebrates, particularly soft corals. However, most studies have targeted the effects of single factors, leaving pronounced knowledge gaps regarding the effects of combined factors on soft corals. Here, we investigated the single and combined effects of phosphate enrichment (1, 2, and 8 μM) and seawater temperature increase (26 to 32 °C) on the soft coral *Xenia umbellata* by quantifying oxygen fluxes, protein content, and stable isotope signatures in a 5-week laboratory experiment. Findings revealed no significant effects of temperature increase, phosphate enrichment, and the combination of both factors on oxygen fluxes. However, regardless of the phosphate treatment, total protein content and carbon stable isotope ratios decreased significantly by 62% and 7% under temperature increase, respectively, suggesting an increased assimilation of their energy reserves. Therefore, we hypothesize that heterotrophic feeding may be important for *X. umbellata* to sustain their energy reserves under temperature increase, highlighting the advantages of a mixotrophic strategy. Overall, *X. umbellata* shows a high tolerance towards changes in global and local factors, which may explain their competitive advantage observed at many Indo-Pacific reef locations.

## Introduction

Coral reefs are highly diverse and complex ecosystems that play a crucial role for humankind as they provide a range of ecosystem services^1,2^. Although coral reefs are ecologically and economically important, they are in decline due to several global and local anthropogenic factors^2,3^. On a global scale, they are affected by increasing seawater temperatures^4^, ocean acidification^5^, and higher ultraviolet radiation^6^. While on a local scale, they may additionally experience factors such as eutrophication in coastal waters^7,8^. This often results in substantial ecological shifts, also termed phase shifts, due to the decline in coral cover, loss of diversity on coral reefs^9^, and general reef degradation^10^. As these global and local factors can impact a coral reef simultaneously, it is important to understand which factors are the main drivers of ecological degradation and possible phase shifts, and how they interact with each other^11^.

Over the last decades, many studies examined the effects of thermal stress^12^ or nutrient enrichment^9,13^ on corals. However, their main focus was on single factors and their impacts on hard coral species. Thermal stress can lead to bleaching of the corals and subsequently reduced photosynthetic capacity^14^ but also a higher rate of mortality^15^, decreased growth^16^, and an increased susceptibility to diseases^17^. Experiments that examined hard corals under nutrient-enriched conditions showed widely varying results, ranging from somewhat positive effects^18–21^ to clear negative impacts^22–24^.

Recently, research is increasingly focussed on the combination of different potential stressors^11,25^. Experiments with combined nutrient enrichment and thermal stress showed divergent results. Experiments by Wiedenmann et al.^26^ showed that hard corals exposed to increased concentrations of dissolved inorganic nitrogen (DIN) were more susceptible to bleaching than corals exposed to low DIN concentrations. This increased bleaching susceptibility may ultimately be caused by a change in the holobionts’ resource partitioning^27^. The endosymbiotic algae of the family Symbiodiniaceae^28^ may retain more photosynthates for its own growth as nitrogen (N) is no longer the limiting nutrient, which is essential for steady translocation of photosynthates from the Symbiodiniaceae to the coral host^29–31^. Consequently, an increase in DIN leads to increasing numbers of algae, which results in phosphorous (P) starvation and in the end to alterations in the thylakoid membranes. This increases the susceptibility of the coral holobiont to thermal and light stress^26^. As in this hypothesis P is the limiting factor, enriching the water with P can potentially prevent bleaching^26,32^. This interruption of N limitation for the Symbiodiniaceae may also be induced by increasing water temperatures^27,32^. As such, P may be of extreme importance for future coral reef health. Since the scientific focus was directed at hard corals, it remains primarily unknown how soft corals are affected by multiple stressors^33^.

In general, soft corals are more resilient to ocean acidification and warming compared to hard corals^34,35^. In addition, many soft corals can rapidly colonize new areas due to their high fecundity and different dispersal modes^36,37^. Given the decrease in hard coral cover on many reefs and reported shifts in benthic reef communities towards non-hard coral taxa^38,39^, it is crucial to understand the ecophysiology of soft corals under different factors^33,35^, and how they may alter the nutrient budgets on coral reefs^40,41^. Particularly successful spreaders are soft corals that belong to the family of Xeniidae. Xeniids are recently being considered as strong invasive species that dominate alternative states on many reefs after phase shifts, like in the Caribbean Sea^42,43^, Indonesia^36,44^, or the southwest Atlantic^45,46^. Few studies, conducted on the soft coral *Xenia umbellata*, showed that glucose enrichment^47,48^ increased, while nitrate enrichment^49^ decreased the corals’ tolerance to thermal stress. Yet, it remains unknown how phosphate (PO_4_) enrichment affects its tolerance to warming.

Therefore, we conducted a 5-week manipulative aquarium experiment to assess the effects of PO_4_ enrichment and warming as single and combined factors on (1) oxygen fluxes, (2) protein content, and (3) elemental and stable isotope composition of the soft coral *X. umbellata*. For this, coral fragments were exposed to three different, ecologically relevant^50^ concentrations of PO_4_ (1, 2, and 8 µM) in combination with warming (26 to 32 °C). The response variables were assessed at different timepoints and finally we put the results in the context of *X. umbellata’s* metabolism and hypothesize what the ecological effects may be.

## Materials and methods

### Experimental design

We conducted a 5-week manipulative aquarium experiment in the Marine Ecology Lab in the Centre for Environmental Research and Sustainable Technology (UFT) at the University of Bremen in Bremen, Germany. The specimens of *X. umbellata* that were used in this experiment are originally from the Red Sea and have been in steady culture in the main holding tank for more than 2 years on a day-night rhythm of 12:12 h (temperature ~ 27 °C, salinity ~ 35 ‰, light ~ 100 µmol photons m^−2^ s^−1^ photosynthetically active radiation (PAR)).

Clonal *Xenia* colonies from the main holding tank were fragmented into 260 smaller colonies of approximately 1–2 cm in width using a scalpel. All colony fragments, at the beginning of the experiment, were similarly sized and roughly contained between 20 and 60 polyps. We then attached these fragments to plugs made of calcium carbonate (AF Plug Rocks, Aquaforest, Poland) using rubber bands. For healing from the fragmentation process, colonies were kept under regular maintenance conditions for 2 weeks. After that, we evenly and randomly distributed the colonies among 12 individual tanks with each individual tank having a total volume of 60 L filled with 40 L of artificial seawater to let them acclimatize to the new environment for 2 weeks before the start of the experiment. Each tank consisted of 2 parts that were interconnected, i.e. a technical part and an experimental part. In the technical part we installed a heater (3613 aquarium heater; 75 W 220–240 V; EHEIM GmbH and Co. KG, Germany) and a recirculation pump (EHEIM CompactOn 1000 pump; EHEIM GmbH and Co. KG, Germany) to ensure constant temperatures and water flow. Each heater was connected to a separate temperature controller (TRD digital, SCHEGO GmbH and Co. KG, Germany) to allow for precise control of the water temperature. Each tank was filled with artificial seawater (Zoo Mix, Tropic Marin, Switzerland) and a layer of CaCO_3_ reef sand to ensure the development of healthy mesocosms. A HOBO Pendant Data Logger (HOBO pendant temp/light, Onset, USA) was placed at the bottom of the experimental part of the tank during the whole experiment to monitor temperature and light conditions (measurements were taken every hour). Coral fragments (*n* = 20 per tank) were then placed on a grid made from eggcrate. Above each experimental part of the tank, we placed two light-emitting diode (LED) lamps (one Royal Blue matrix module and one Ultra Blue White matrix module, WALTRON daytime LED light, Germany) so that light levels were similar to conditions in the maintenance aquarium (~ 100 µmol photons m^−2^ s^−1^ PAR) on a day-night rhythm of 12:12 h. Light intensities were tested twice a week with a LI-1400 Data Logger (LI-COR Biosciences GmbH, Germany) and adjusted when necessary.

To ensure stable conditions in the tanks, 10% daily water exchanges were done to mimic the high renewal rate of seawater that can be found on coral reefs. Due to the daily water exchanges we did not install additional protein skimmers. Water parameters were maintained at the following levels (mean ± SD): salinity 36.1 ± 0.3 ‰, nitrate < 0.5 mg L^−1^, ammonium < 0.05 mg L^−1^, nitrite < 0.01 mg L^−1^, calcium 435 ± 150 mg L^−1^, alkalinity 7.3 ± 2.5°dH, magnesium 1495 ± 514 mg L^−1^, and pH 8.6 ± 0.2. These water parameters are equal to the ones the corals previously experienced over the last 2 years in the main holding tank filled with artificial seawater, with the elevated pH value likely caused by a high animal to water ratio. Coral plugs were cleaned from biofouling twice per week. Also, we fed the corals with 0.1 g of dried marine plankton (Reef-Roids, Polyp Lab, USA) per tank twice a week to avoid stress from starvation and to mimic conditions from the main holding tank. Yet, in the main holding tank corals lived in an ecosystem also containing fish and other organisms, hence in the holding tank they may have benefited also indirectly from the fish feed entering the water column which was missing in our experimental setup.

### Experimental phosphate and temperature treatments

**Figure 1:**
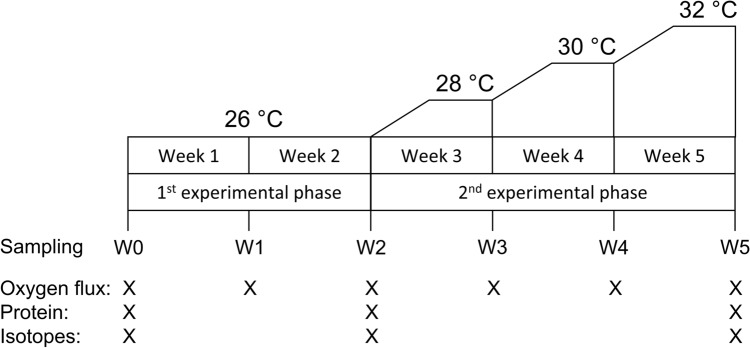
Experimental design with a 1st experimental phase of pure PO_4_ enrichment, followed by a 2nd experimental phase during which temperature was increased stepwise from 26 to 32 °C. Sampling of oxygen fluxes took place after each week (W0-5), while corals for protein content as well as elemental and stable isotope analysis were collected at the beginning, and at the end of each experimental phase.

We created three different PO_4_ enrichment treatments (1, 2, and 8 µM), which are comparable to previously conducted experiments with corals in the lab and the field^23,24,51–54^ and a control treatment without PO_4_ addition (*n* = 3 tanks per treatment). For the first 14 days of the experiment, the corals were exposed to PO_4_ enrichment only (Fig. 1). The length of the pure eutrophication treatment was chosen (1) as a previous study could already detect an effect of PO_4_ and nitrate (NO_3_) after 2 weeks^55^, and (2) to allow for a better comparison due to similar experimental design of closely related studies conducted on *X. umbellata*^47–49^. To keep the PO_4_ enrichment treatments stable, we measured PO_4_ concentrations every day after the water exchange with an adjusted protocol of a commercially available PO_4_^3−^ test kit for salt water (TESTLAB MARIN, JBL, Germany) using a photometer (Turner Designs Trilogy Laboratory Fluorometer). For this we quantified weights and volumes of reagents and created a calibration curve (R^2^ = 0.967). Afterwards PO_4_ concentrations were manually adjusted in each tank using a 1 M stock solution from sodium phosphate dibasic Dihydrate (Na_2_HPO_**4**_ × 2 H_2_O).

On day 15, we started the second part of the experiment with the stepwise ramping of the temperature up to 32 °C (Fig. 1). For this, the temperature was increased by 1 °C day^−1^ on 2 consecutive days and then kept constant for 5 days. We conducted such a 7-day cycle three times until the temperature reached 32 °C. The temperature treatment in our laboratory experiment were synonymous to 2 degree heating weeks (DHW)^56^ in the first, 6 DHW in the second, and 12 DHW in the third week of the 2nd phase of the experiment.

### Oxygen flux measurements

One fragment per tank (*n* = 3 per treatment) was labeled and used throughout the experiment to determine the rates of oxygen consumption in the dark (dark respiration, R) and oxygen production in the light (net photosynthesis, P_net_). The smallest colony tested for oxygen fluxes consisted of 28 polyps, while the biggest colony consisted of 70 polyps. To avoid stress, we transferred the colonies without air exposure to incubation chambers with a volume of 160 mL. We filled the incubation chambers with water from the respective tank and sealed them gas-tight without air bubbles inside. Then, we placed them in a temperature bath on a magnetic stirrer (Thermo Scientific, Variomag Poly) with 190 rotations per minute (rpm) for 1.5 to 2 h in the light and dark. The stirring bars allowed for mixing of the water column and ensured homogenous oxygen concentrations in the chambers throughout the measurement. The light used to measure oxygen production was emitted with approximately 100 µmol photons m^−2^ s^−1^ PAR by an LED light (Royal Blue—matrix module and Ultra Blue White 1:1—matrix module, WALTRON daytime LED light, Germany) to ensure equal light conditions as in the experimental tower.

We measured O_2_ concentrations within each chamber before and after each incubation by an optode sensor (HACH LDO, HACH HQ 40d, Hach Lange, Germany). For later analysis, we calculated P_net_ in the light, and R in the dark, accounted for water background metabolism, and normalized the flux by incubation duration, chamber volume, and the corals’ surface area (SA), to account for the differences in coral fragment size. This is common procedure when calculating oxygen fluxes and has been done in many studies beforehand^57–59^, with studies conducted on *X. umbellata* reporting oxygen fluxes in the unit of mg O_2_ m^−2^ h^−1^^47,49^. The calculations of the SA per polyp were based on the geometric method developed by Bednarz et al.^52^, multiplying the average polyp SA with the number of polyps. We additionally calculated gross photosynthesis (P_gross_) rates, based on the assumption that respiration is constant during the day. Yet, as respiration rates may be significantly higher during active photosynthesis than in the dark^60^, the calculated P_gross_ rates can be seen as conservative estimates based on dark respiration. Running a linear regression on the log-transformed data, we analysed that both P_gross_ and R were negatively allometric to the number of polyps with a slope of 0.34 and 0.50, respectively. This indicates that larger colonies produce and consume more oxygen, but relatively less oxygen is produced and consumed per polyp. Therefore, two hypotheses arise: (1) the ratio of coral base to polyp number of coral fragments with many polyps may be smaller than the ratio of colonies with fewer polyps, and is not accounted for by the method of Bednarz et al.^52^, and (2) self-shading in bigger colonies could also potentially cause non-isometric scaling.

### Protein content measurements

For measuring the total protein quantification, we used the Bradford assay^61^ following the Coomassie Protein Assay Kit (Thermo Scientific). For this, we took one *X. umbellata* fragment out of each tank (*n* = 3 per treatment) randomly each week, rinsed it in distilled water to remove salt, and stored it in a plastic bag in the freezer at − 20 °C until further processing. We lyophilized these colonies for 24 h at − 60 °C and stored them under dark and dry conditions pending analysis. During lyophilization the samples get freeze-dried by sublimating all the liquid from the sample, leaving only the dry compounds of the animal, e.g., protein and carbohydrates, behind. After that, we then ground those dried samples using mortar and pestle and measured the dry weight (DW) of each colony to use as a normalization metric. The DW of the colonies ranged from 12 to 97 mg (see Supplementary Material). We used this to standardize the protein content of each colony. Using a linear regression on the log-transformed data of protein content and DW, we found a strong correlation between protein content and DW (R^2^ = 0.80, see Supplementary Fig. S3). The slope above 1 indicates a positive allometry with protein content increasing faster than colony size.

After this, we homogenized the powder from every single sample in 5 mL of distilled water using a high-speed homogenizer (VEVOR, FSH-2A) for 2 min to extract the protein. For further quantification of the protein, we loaded 1 mL from each sample and mixed it with 1 mL of Bradford Dye Reagent 1× (Coomassie Brilliant Blue) in cuvettes. Lastly, we incubated the samples at room temperature for 10 min and read the absorbance in the spectrophotometer under 595 nm wavelength. To gain the resulting concentration of protein in the water we calculated it via the measured absorbance, using a diluted Bovine Serum Albumin (BSA) calibration curve, and then standardized it to water volume and coral dry weight.

### Carbon and nitrogen elemental, and stable isotope analysis

To assess carbon (C) and nitrogen (N) isotope signatures and C:N ratios of *X. umbellata*, we conducted an elemental and stable isotope analysis on the entire holobiont. Every week on the first day we randomly selected one fragment of each tank (*n* = 3 per treatment), counted polyps, removed the colony from the plug, washed it in distilled water to remove salt, stored it in a plastic bag, and froze the sample at − 20 °C until further processing. Upon processing, *X. umbellata* colonies were dried in sterile glass petri dishes at 40 °C until weight consistency was reached (~ 48 h). Then, we ground the dried colonies into a fine powder using mortar and pestle, weighed the tissue powder, and transferred 1–2 mg into tin cups. Samples were analysed for C and N quantities as well as stable isotope ratios as described in Karcher et al.^40^. Isotopic ratios (r) are given as the ratio of the heavier to the lighter isotope (^15^N:^14^N or ^13^C:^12^C) and notated as either δ^15^N or δ^13^C (‰) using

1 $$\delta X=\left(\frac{{r}_{sample}}{{r}_{reference}}-1\right)\times 1000,$$

where r_reference_ for δ^15^N is atmospheric N (0.00368) and Vienna Pee Dee Belemnite (0.01118) for δ^13^C.

By analysing the corals’ elemental and stable isotope composition, it is possible to infer the sources of C and N in the coral holobiont. While carbon stable isotope ratios (δ^13^C) are often used to understand the corals’ C-metabolism^55^, the N stable isotope composition (δ^15^N) can be used as a bioindicator to determine the N source of the holobiont^62^. This can be done as different sources for C and N result in different stable isotope ratios. While C fixed by the Symbiodiniaceae is mostly comprised out of dissolved inorganic carbon (DIC) from the surrounding seawater (δ^13^C DIC = 0‰)^63,64^, C acquired via heterotrophic feeding on plankton has a different δ^13^C value (e.g. δ^13^C zoop =  − 19‰ or more negative^65,66^). Therefore, δ^13^C values vary proportionately to the contribution of either photosynthates or heterotrophic feeding as the main C source for the coral^67–72^. The same applies to δ^15^N values, which are used to determine whether the corals are getting their N mainly from dinitrogen (N_2_) fixation of atmospheric N_2_ (δ^15^N = 0‰)^73^, which decreases the values^74^. Or if the coral is getting its N from other sources like sewage- and tourism-derived nutrient loading^75^, which increases the value with increasing uptake of anthropogenic N^62,76^.

### Statistical analysis

We carried out the statistical analysis using RStudio (Version 1.4.1106)^77^ with the packages tidyverse^78^, ggpubr^79^, and rstatix^80^. As we used three separate tanks per treatment, we did not have a nested design and assume the tank effect to be zero for all statistical analyses. Additionally, all data was tested for possible tank effects by inspecting it for significant differences between tanks in all measured water parameters, but none were detected. To test for significant effects of treatments over time in invasive parameters (protein content and elemental stoichiometry) we used the 2-way analyses of variance (ANOVA). Log-transformation of ratios was not conducted as the data was normally distributed. We tested repeated measurements (Pgross, and R) using a 2-way mixed ANOVA with ‘Time’ as within-subject factor and ‘Treatment’ as between-subject factor. First, we tested data sets for outliers and then tested normal distribution of the data using the Shapiro Wilk test and additionally qqplots for visual confirmation. We conducted Levene’s test to test for homogeneity of variances, while we used Box’s M-test for homogeneity of covariances. Thereby, sphericity was automatically tested with Mauchly’s test and corrected when violated using the Greenhouse–Geisser sphericity correction. Lastly, we did a post-hoc analysis using pairwise comparison tests with Bonferroni adjustment with t-tests, and Dunn’s test for non-parametric data. We considered results to be significant with a p-value lower than 0.05 (*p* < 0.05) and display them as mean ± standard deviation. All raw data is given in the Supplementary Material online.

## Results

### Effects on oxygen fluxes

Compared to baseline values both, gross photosynthesis (P_gross_) values and respiration (R), remained stable under PO_4_ enrichment and temperature increase, alone and combined. The growth of investigated coral colonies was around 0.14–0.30 polyps per day without any significant differences between treatments.

**Figure 2:**
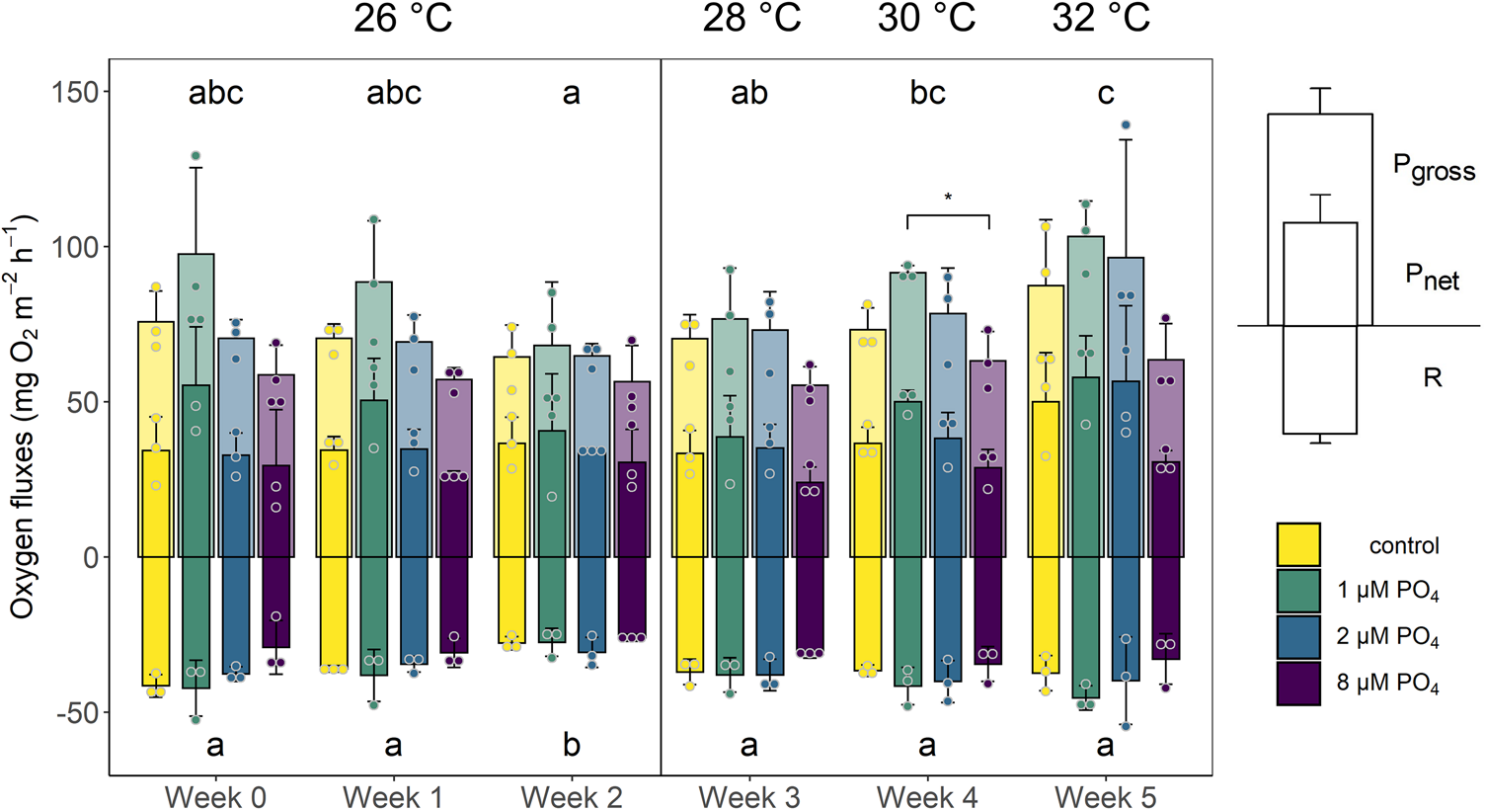
Gross- (P_gross_), net photosynthesis (P_net_), and respiration (R) of *Xenia umbellata* under experimental conditions: only temperature increase without PO_4_ addition (control), 1 µM PO_4_ + temperature increased from week 3 onwards (1 µM PO_4_), 2 µM PO_4_ + temperature increased (2 µM PO_4_), and 8 µM PO_4_ + temperature increased (8 µM PO_4_). Letters indicate significant differences for P_gross_ and R between weeks and asterisks indicate significant differences between treatments per time (p < 0.05, pairwise comparison t-test, Bonferroni adjustment). Error bars represent standard deviations. Dots represent the individually measured data points. The vertical line indicates the start of the temperature treatment and the average temperature for all tanks for the different time points is given on top of the graph.

Under ambient conditions, *X. umbellata* colonies exhibited stable oxygen fluxes with P_gross_ and R values averaging 74 and − 36 mg O_2_ m^−2^ h^−1^, respectively. Though the overall effect of PO_4_ enrichment on P_gross_ values (Fig. 2) was not significant (2-way mixed ANOVA, F(3, 8) = 2.987, *p* = 0.096), there was a significant difference between the P_gross_ values of colonies in the low and high PO_4_ treatment in week 4 (pwc, Bonferroni adjustment, t-test, *p* = 0.0386). Additionally, P_gross_ values for all treatments varied significantly over time (2-way mixed ANOVA, F(5, 40) = 8.538, *p* < 0.001) with highest values after 5 and lowest values after 2 weeks. When temperatures increased to 30 °C, colonies from the low PO_4_ treatment had a P_gross_ rate 31% higher than colonies from the high PO_4_ treatment (91.5 and 63.2 mg O_2_ m^−2^ h^−1^, respectively). During the remaining days of the experiment, no significant treatment effects were observed, and all P_gross_ values were stable compared to baseline measurements throughout the experiment.

Respiration, just like P_gross_ values, was unaffected by PO_4_ enrichment, as controls were not significantly different from PO_4_ treated colonies, but showed significant differences over time (2-way mixed ANOVA, F(1.34, 10.69) = 9.308, *p* = 0.008) with respiration rates being the lowest after 2 weeks of pure PO_4_ enrichment (Fig. 2). However, while respiration increased during the stepwise temperature increase compared to values from week 2, with the highest values in weeks 4 and 5 (− 38.215 and − 38.899 mg O_2_ m^−2^ h^−1^, respectively), it did not significantly change compared to baseline values (− 37.648 mg O_2_ m^−2^ h^−1^).

### Effects on protein content

Protein content did not change between different PO_4_ enrichment treatments but changed over time and with increasing temperatures.

**Figure 3:**
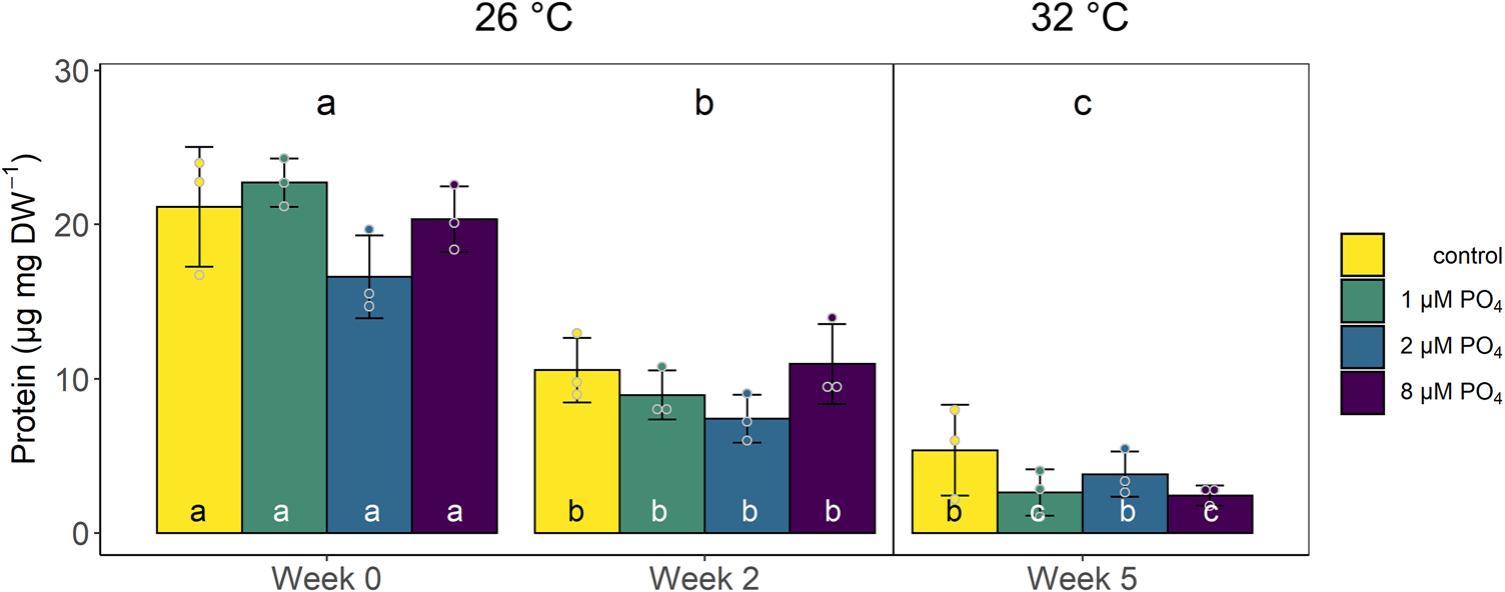
Protein content of *Xenia umbellata* colonies under experimental conditions: only temperature increase without PO_4_ addition (control), 1 µM PO_4_ + temperature increased from week 3 onwards (1 µM PO_4_), 2 µM PO_4_ + temperature increased (2 µM PO_4_), and 8 µM PO_4_ + temperature increased (8 µM PO_4_). Letters above bars indicate significant differences between weeks and letters within bars indicate significant differences within the treatment over time (p < 0.05, pairwise comparison t-test, Bonferroni adjustment). Error bars represent standard deviations. Dots represent the individually measured data points. The vertical line indicates the start of the temperature treatment and the average temperature for all tanks for the different time points is given on top of the graph.

Protein content in *X. umbellata* colonies (Fig. 3, Supplementary Fig. S3) changed significantly between PO_4_ enrichment (2-way mixed ANOVA, F(3,24) = 3.076, *p* = 0.047) and over time (2-way mixed ANOVA, F(2,24) = 174.187, *p* < 0.001). The highest values were found in the baseline measurement with on average 20.2 µg protein mg^−1^ coral DW. After 2 weeks of PO_4_ enrichment alone, protein content significantly decreased by more than 50% to 9.48 µg protein mg^−1^ coral DW (pwc, Bonferroni adjustment, t-test, *p* < 0.001), and after the additional temperature increase, protein content dropped further down to 3.56 µg protein mg^−1^ coral DW, which differed significantly from the two previous measurements (pwc, Bonferroni adjustment, t-test, *p* < 0.001). While there were no significant differences between PO_4_ treatments at each time point, there were highly significant differences in each treatment over time, with the highest values always at the start and lowest values at the end of the experiment. Only the control and medium PO_4_ treatment corals showed no further significant decline in protein content during the additional temperature increase in the last 3 weeks of the experiment.

### Effects on stable isotope signatures

All tested stable isotope signatures remained stable between different PO_4_ enrichment treatments throughout the experiment and were only significantly affected by time and the stepwise temperature increase.

**Figure 4:**
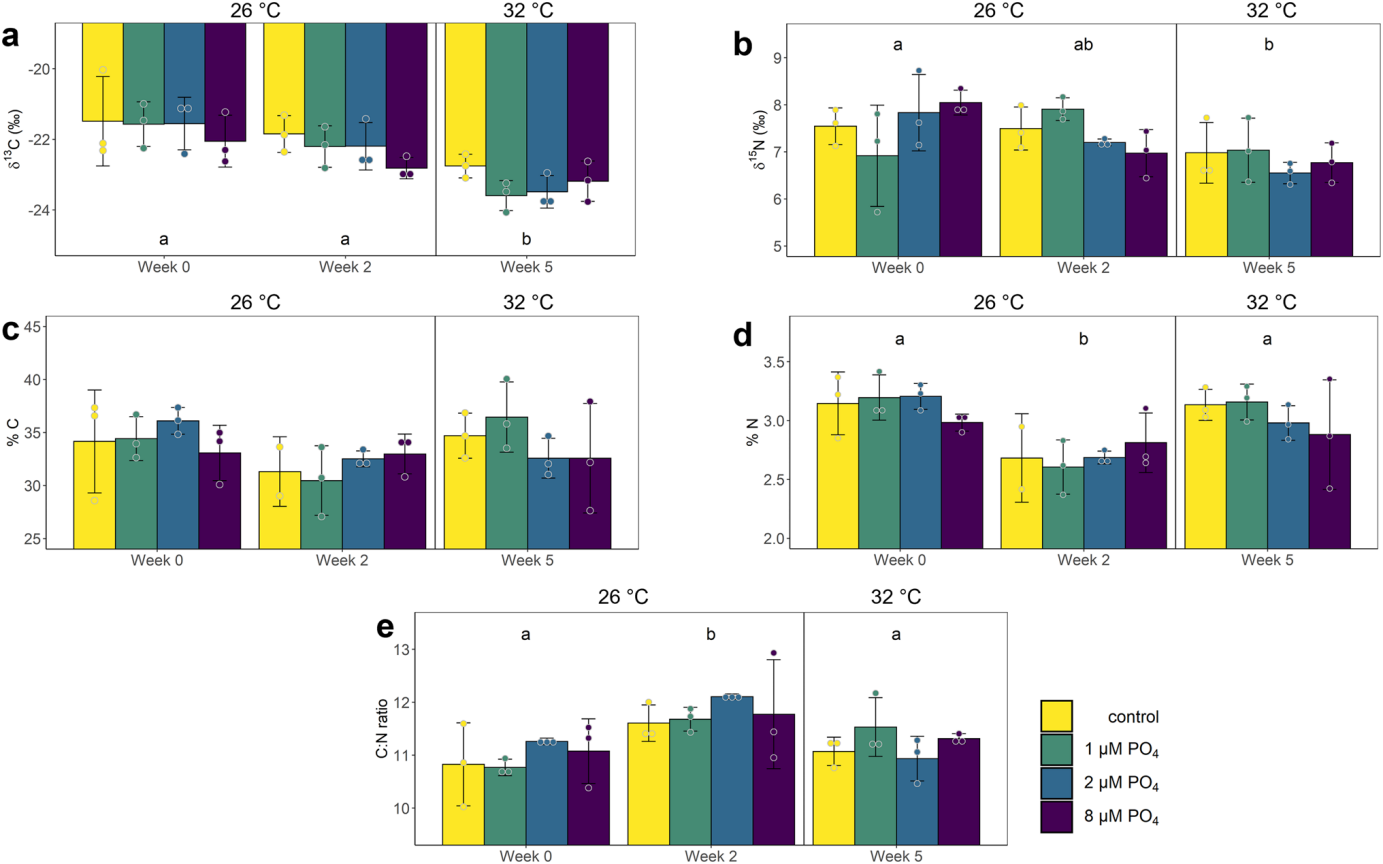
Carbon (**a**), and Nitrogen (**b**) stable isotope ratio, percent carbon (**c**), percent nitrogen (**d**), and carbon to nitrogen ratio (**e**) of *Xenia umbellata* colonies under experimental phosphate conditions: only temperature increase without PO_4_ addition (control), 1 µM PO_4_ + temperature increased from week 3 onwards (1 µM PO_4_), 2 µM PO_4_ + temperature increased (2 µM PO_4_), and 8 µM PO_4_ + temperature increased (8 µM PO_4_). Letters indicate significant differences between weeks (p < 0.05, pairwise comparison t-test, Bonferroni adjustment). Error bars represent standard errors. Dots represent the individually measured data points. The vertical line indicates the start of the temperature treatment and the average temperature for all tanks for the different time points is given on top of the graph.

The δ^15^N, as well as δ^13^C values, changed significantly over time (2-way ANOVA, δ^15^N: F(2,24) = 5.957, *p* = 0.008; δ^13^C: F(2,24) = 18.212, *p* < 0.001) (Fig. 4). While δ^15^N values (Fig. 4A) stayed constant at a level of 7.5 ‰ within the first 2 weeks of pure PO_4_ enrichment, they decreased significantly by 10% over the stepwise temperature increase compared to the baseline (pwc, Bonferroni adjustment, Dunn’s test, *p* = 0.0112). The δ^13^C values (Fig. 4C) also decreased significantly during the temperature increase by 6.9 and 4.3% compared to baseline and the values after 2 weeks of PO_4_ treatment (*p* < 0.001 and *p* = 0.0018, respectively).

The C content of the coral colonies showed no significant differences, while percent N content significantly changed over time (2-way ANOVA, F(2,23) = 11.212, *p* < 0.001) (Fig. 4B,D). The values decreased by 14% within the first 2 weeks of the experiment compared to the baselines (*p* < 0.001) and returned close to starting values of 3.1% N again after the stepwise temperature increase. These differences in percent N content then lead to a significant difference in the total C:N ratio over time (2-way ANOVA, F(2,24) = 8.863, *p* = 0.001) (Fig. 4E). The C:N ratio after 2 weeks of PO_4_ enrichment was on average 6.9% higher and significantly different from the ratios at the start and end of the experiment (*p* < 0.001 and *p* = 0.0137, respectively).

## Discussion

While hard coral cover decreases on reefs worldwide^81–85^, a shift to other benthic organisms, such as soft corals, can be observed^38,39^. As such, we tested the (combined) effects of two potentially stressful factors, i.e., PO_4_ enrichment and warming, on the pulsating soft coral *X. umbellata*. Our results suggest a high tolerance of *X. umbellata* towards increasing temperatures, regardless of PO_4_ enrichment.

### How does PO_4_ enrichment affect *X. umbellata*?

In general, no significant differences between *X. umbellata* in the control and PO_4_ treatment groups were observed throughout the experiment, showing that PO_4_ had no measurable effect on the coral in the observed parameters. Compared to hard corals, soft corals have lower photosynthetic productivity, observable in their respective P_gross_/R ratio of 1.0–1.3, while hard corals range within a P_gross_/R ratio of 2–4^60,86^. Light intensity may have played a role, too. Mergner and Svoboda^86^ measured oxygen fluxes of soft and hard corals from the same location, thereby reducing the effect of light intensity as far as possible. Also, Fabricius and Klumpp^60^ acknowledged this possible limitation when comparing oxygen fluxes from different studies in their discussion. When comparing the absolute values for P_gross_ and R of studies on *X. umbellata*, P_gross_ values reach 22 up to 140 mg O_2_ m^−2^ h^−1^, while respiration ranges between − 11 and − 60 mg O_2_ m^−2^ h^−1^. Despite this range, with a P_gross_/R ratio of around 2 measured in this study and previously^47,49,52^, *X. umbellata* has higher photosynthetic productivity compared to other soft corals but is still at the lower edge of productivity compared to hard corals. The measured oxygen fluxes in our experiment remained stable under all added PO_4_ concentrations. These results are in contrast with a study by Bednarz et al.^52^, which showed increased gross photosynthesis in xeniids under PO_4_ enrichment, most likely due to increased chlorophyll *a* (chl *a)* concentrations. Our results also show that throughout the experiment the protein content significantly decreased in all colonies, regardless of PO_4_ enrichment. Particularly the decrease in protein content over the first 2 weeks without warming was surprising, as the corals have not faced a specific stressor and did not show increased respiration rates, which would indicate an increased energy demand. While *X. umbellata* can live autotrophically^87^, it is a heterotrophic suspension feeder^88^. So, this reduction in protein content indicates that *X. umbellata* metabolised its protein energy reserves, probably as a result of reduced heterotrophy during the experiment, as they were only fed twice a week with dried zooplankton.

### How does PO_4_ enrichment affect the response of *X. umbellata* to a temperature increase?

#### Warming does not disrupt oxygen fluxes in *X. umbellata*

While direct evidence showing that *X. umbellata* experiences PO_4_ starvation under warming scenarios, as hypothesized for hard corals by Wiedenmann et al.^26^, is missing in our study, we here show that PO_4_ enrichment does not affect the corals tolerance towards warming. Overall, the measured oxygen fluxes in our experiment and respectively the P_gross_/R ratio remained stable around 2 in response to warming, regardless of PO_4_ enrichment. The P_gross_/R ratio above 1 indicates that *X. umbellata* can sustain its energy needs via net autotrophy and does not necessarily rely on heterotrophic feeding to meet their daily metabolic demands^89^. These results are surprising at first, as warming increases the respiration of hard corals as a sign of stress and increased energy demand^27,90^. At the same time, a general decrease in photosynthesis was often observed when temperatures exceeded 31 °C, due to a damaged photosystem II during temperature stress^14,91,92^ or as a result of decreased Symbiodiniaceae density or their chl *a* concentrations^93^. A possible explanation for the constant photosynthesis rates observed in the present study may be the special characteristic of *X. umbellata* being a pulsating coral^94^. Pulsation is known to have a positive effect on the corals’ photosynthetic activity, as it enhances gas exchange, leading to a greater efflux of oxygen from the coral tissues and increases the photosynthetic activity^35,94^. Additionally, the constant water movement may alleviate the coral from high temperature stress as fresh seawater is constantly circulating around the polyps, avoiding the build-up of a heated boundary layer. This may have enabled the corals used in the present study to sustain their photosynthetic efficiency even under warming scenarios. An experiment observing the effects of NO_3_ enrichment under warming on *X. umbellata*^49^ found that NO_3_ enrichment decreased P_gross_ rates, possibly due to the reduced pulsation of the polyps. Lastly, Simancas-Giraldo et al.^47^ found that *X. umbellata* colonies exhibit both reduced P_gross_ and R under warming, regardless of additional glucose enrichment. The authors argued that the reduced respiration rates may be a result of an explicit metabolic depression.

#### Warming leads to a significant decrease in protein content, δ15N, and δ13C

The protein content continued to significantly decrease over time with increasing temperature. This may contradict other studies, which showed that water temperature did not affect the protein content of the hard corals *Stylophora pistillata*^95^ and *Turbinaria reniformis*^93^. Yet, it could also be an effect of time and not temperature specifically, as protein content already significantly declined without increased temperatures, showing that *X. umbellata* metabolizes its energy reserves in the experimental setup. Additionally, total N content went back to baseline values under increased temperatures, regardless of PO_4_ treatment. A possible explanation could be that the higher temperatures led to increased N_2_ fixation, as shown in several studies on hard corals^96–99^, and therefore higher N availability and incorporation of it in the tissue of the holobiont. This hypothesis is supported by the decrease in δ^15^N as this could be a sign of increased N_2_ fixation^74^. Increased N_2_ fixation may relieve the Symbiodiniaceae from N-limitation. In combination with retaining their photosynthates for themselves, these conditions may allow the Symbiodiniaceae to grow, leaving the host with less organic C, as demonstrated for the hard coral species *Stylophora pistillata*^27^. This hypothesis is supported by the parallel study of Klinke et al.^100^, who showed increased algal cell densities under higher temperatures. However, a recent study by Rädecker et al.^101^ shows that diazotrophs derived N may not be utilized by the Symbiodiniaceae under heat stress. Yet, as the coral is catabolizing its protein reserves, N is also released and becomes available. As *X. umbellata* seems to be rather tolerant toward increased temperatures, the alleviation from N-limitation may be due to N_2_ fixation, or N from the proteins, or a combination of both.

In the present study, both δ^15^N and δ^13^C significantly decreased from the start of the experiment until the end after 5 weeks of PO_4_ enrichment and additional warming, regardless of the PO_4_ treatment. A decrease in δ^13^C is often a sign that less photosynthates are shared with the host organism who then, in turn, relies more on heterotrophic feeding, as zooplankton is depleted of the heavier ^13^C isotope^102,103^. However, in our study, we did not observe a change in oxygen fluxes, therefore fewer photosynthates are unlikely to be the reason for this decrease. A possible explanation could be that (1) the corals preferentially catabolized isotopically heavier organic matter, therefore reducing the δ^13^C value^55^. For example, bleached corals have reduced photosynthesis and catabolize heavier-isotope lipids, which overall depletes the lipid δ^13^C^70^. In the present study, the corals gradually lost their protein content, suggesting that at the same time isotopically heavier lipids may have been catabolized. Tanaka et al.^55^ suggest the same pathway as an explanation for the depletion of δ^13^C in their corals experiencing pure NO_3_ enrichment, implying that corals under imbalanced N:P ratios consume more lipids than corals under balanced N:P ratios. Another possible explanation for a decrease in δ^13^C may be (2) a net release of Symbiodiniaceae derived photosynthates into the water column, thereby shifting the δ^13^C value. While the results of measured total organic carbon (TOC) concentrations in the water during the incubation for oxygen fluxes were obscured by high standard deviation and background fluxes, a net release of TOC was observed for colonies under PO_4_ enrichment (see Supplementary Fig. S1). Lastly, an alternative explanation could be (3) the metabolization of the added marine zooplankton (reef roids) in the tanks’ sediment. Thereby, the reef roids may have altered the isotopic composition of the available CO_2_ in the tank which was then used by the corals for photosynthesis thereby causing the more negative δ^13^C through a secondary pathway.

### Ecological implications

Despite soft corals’ growing importance due to phase shifts and their increasing abundance on reefs worldwide, there is a knowledge gap on how the simultaneously occurring alterations in the seawater N:P ratio and ocean warming scenarios affect soft coral species. Since 64% of all reefs are located near the shoreline of densely populated areas, they are more likely to be exposed to high inputs of inorganic nutrients in the water from human activities^26,104,105^. Groundwater percolation and submarine groundwater discharge is likely a major source of nutrient input on reefs^50,106,107^. Combined with ocean warming, this can have detrimental effects on hard corals^26,108^.

Compared to studies conducted with hard corals looking at either nutrient enrichment or temperature increase (Supplementary Material Table S1), it becomes clear that *X. umbellata* is relatively unaffected by these factors. In Table S1 it is shown that warming above 2.5 °C led to lowered P_gross_, increased R and consequently reduced P/R ratios in 85% of species observed. Also, Fv/Fm, a proxy of photosynthetic activity, decreased in 6 of 7 observed species. Additionally, protein content was reduced in half of the observed species, indicating a need for using energy reserves under increased temperatures. While generally less studies focus on the effects of PO_4_ enrichment compared to temperature increase, only two studies combined these two factors. One of these two studies showed that *Stylophora pistillata* had reduced Fv/Fm and P_net_ under temperature increase and nutrient enrichment, respectively, leaving the authors to hypothesize that eutrophication may compromise the corals resilience to global change^109^. While the other study showed that *Pocillopora damicornis* had increased R under warming, even more pronounced when combined with PO_4_ enrichment, and decreased P_gross_ rates^54^.

With *X. umbellata* being able to live autotrophically^87^, and not showing any changes in P_gross_ and R values under warming and PO_4_ enriched conditions, this would imply their resistance towards these two factors, even under extended periods. Nevertheless, our study indicates that *X. umbellata* had to utilize its energy reserves during the course of the experiment. Thobor et al.^49^ found a significant decrease in P_gross_ of *X. umbellata* under nitrate enrichment, but no significant changes under temperature increase. The combination of both factors led to severe declines in polyp pulsation, increased tissue loss, and mortality. This indicates that while *X. umbellata* is resistant towards warming, nitrate enrichment may reduce this resistance, potentially by disrupting the symbiosis between the coral host and its Symbiodiniaceae, as was found for hard corals^26^. This scenario would lead to a higher dependence of the coral on heterotrophic feeding.

While hard corals from the northern Red Sea show a net release of particulate organic matter (POM)^110^, *X. umbellata* showed net POM uptake^52^. Such coral-derived POM plays an essential role in the recycling of nutrients in reef ecosystems^111^. With *X. umbellata* not contributing to POM release, but instead actively taking up the organic matter, this may have implications for reef C cycling under shifts towards soft coral dominance due to their invasiveness. But not only C cycling may change in soft coral dominated reefs, but also N cycling. In this context, a study of El-Khaled et al.^41^ speculated that soft corals, i.e. *Xenia* sp., can help alleviate reefs from excessive N, as they may play a key role in reef-wide denitrification. Future studies should therefore conduct further investigations into the metabolism of *X. umbellata* to help clarify under which scenarios this functional autotroph changes to a mixotrophic strategy, to what extent, and how this may affect overall energy and nutrient budgets on future reefs.

## Supplementary Information

Supplementary Figures.

Supplementary Table S1.

## Data Availability

Raw data of the current study is available in the Supplementary Material online.
