# Supplementary material for: The widely distributed soft coral Xenia umbellata exhibits high resistance against phosphate enrichment and temperature increase
Source: Sci Rep. 2022 Dec 22;12:22135. doi: 10.1038/s41598-022-26325-5 (PMC9780247; doi:10.1038/s41598-022-26325-5)
Supplement: Supplementary file 1 — Supplementary Figures. [file 41598_2022_26325_MOESM1_ESM.pdf]

The widely distributed soft coral *Xenia umbellata* exhibits high resistance against phosphate enrichment and temperature increase

- Supplementary Material -

Selma D. Mezger*[1], Annabell Klinke[1,2], Arjen Tilstra[1], Yusuf C. El-Khaled[1], Bianca Thobor[1], Christian Wild[1]

[1]University of Bremen, Faculty of Biology and Chemistry, Department of Marine Ecology, Leobener Str. 6, 28359 Bremen, Germany

[2] Leibniz Centre for Tropical Marine Research, Fahrenheitstraße 6, 28359 Bremen, Germany

*corresponding author: Selma D. Mezger, mezger@uni-bremen.de

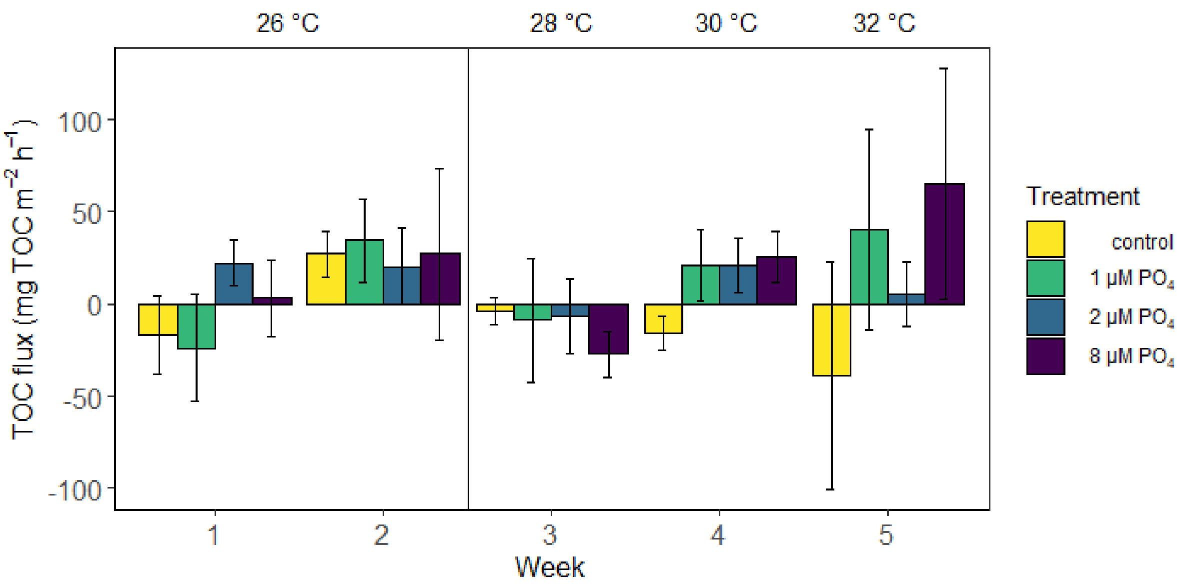

Figure S1. TOC fluxes of *Xenia umbellata* colonies under experimental conditions: only temperature increase without PO_4 addition (control), 1 µM PO_4 + temperature increased from week 3 onwards (1 µM PO_4), 2 µM PO_4 + temperature increased (2 µM PO_4), and 8 µM PO_4 + temperature increased (8 µM PO_4). Error bars represent standard errors. The vertical line indicates the start of the temperature treatment and the average temperature for all tanks for the different time points is given on top of the graph.

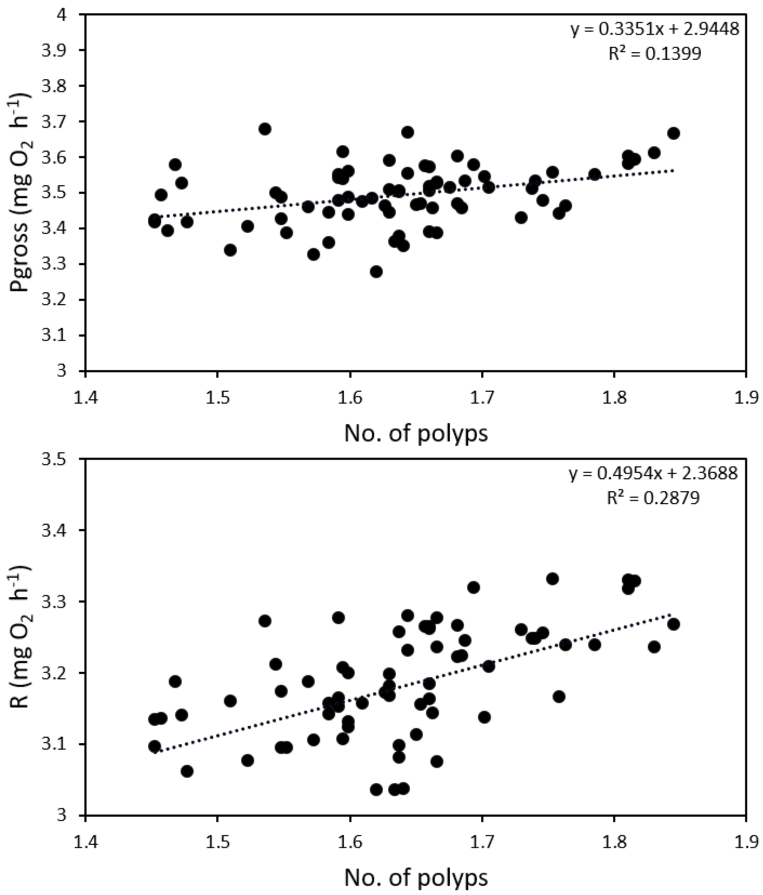

Figure S2. Correlation of log-transformed oxygen fluxes and number of polyps in *Xenia umbellata* colonies including all treatments.

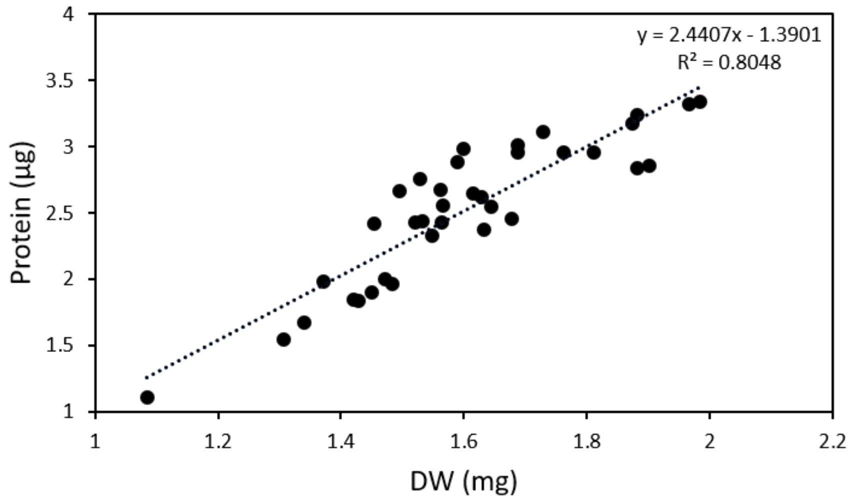

Figure S3. Correlation of log-transformed protein content and dry weight in *Xenia umbellata* colonies including all treatments.

Table S1. Experimental studies on effects of phosphate enrichment (PO_4) and temperature increase (Temp.) on hard corals in comparison to the present study. Only parameters similar to the ones measured in the present study were summarized. N = nitrogen source, P = phosphate source, P_{gross} = gross photosynthesis, P_{net} = net photosynthesis, R = dark respiration, P/R = photosynthesis to respiration ratio

Study	Duration	Treatment	Coral species	Parameter	Effect
EUTROPHICATION EXPERIMENTS					
Ferrier-Pagès et al. 2000 [1]	9 weeks	2 µM PO_4	*Stylophora pistillata*	growth	Decrease (- 60 %)
				P_{gross}	Increase (+ 100 %)
				R	Increase (+ 59 %)
				P/R	Increase (+ 47)
Koop et al. 2001 [2]	406 days	20 µM Ammonium and/or 4µM PO_4	*Stylophora pistillata*	P_{gross}	P: decrease (- 19 %)<br>N+P: decrease (- 14 %)
				R	n.s.
Stambler et al. 1991 [3]	28 days	0.5 and 2 µM PO_4	*Pocillopora damicornis*	Protein	n.s.
Tanaka et al. 2017 [4]	2 months	Nitrate: 1.4-1.9 µM<br>PO_4: 0.1 µM	*Montipora digitata*	Symbiont C content	N: n.s.<br>N+P: Increase (+ 48 %)
				Symbiont N content	N: Increase (NA)<br>N+P: Increase (NA)
				Host C + N content	n.s.
				Host and Symbiont δ13C	N: Decrease (Host: - 6 %, Symbiont: - 5 %)<br>N+P: n.s.
Hoegh-Guldberg et al. 2004 [5]	406 days	20 µM Ammonium and/or 4µM PO_3^{2+}	*Heliofungia actiniformis*	Host δ15N	P: Decrease (- 68 %)<br>N+P: Decrease (- 47 %)
				Symbiont δ15N	P: n.s.<br>N+P: Decrease (- 55 %)
				δ13C	n.s.
			Pocillopora damicornis	Host δ15N	P: n.s.<br>N+P: Decrease (- 55 %)
				Symbiont δ15N	P: n.s.<br>N+P: Decrease (- 73 %)
				δ13C	n.s.
Silbiger et al. 2018 [6]	6 weeks	medium (3.6 µmol l^{-1} DIN + 1.08 µmol l^{-1} PO_4); high (7.61 µmol l^{-1} DIN + 2.6 µmol l^{-1} PO4)	*Porites compressa and Montipora caiptata*	R	Medium: Increase (+ 7 %)<br>High: Increase (+ 51 %)
TEMPERATURE EXPERIMENTS					
Krueger et al. 2017 [7]	6 weeks	1-2 °C	*Stylophora pistillata*	P_{net}	Increase (+ 44 %)
				Protein	n.s.
Schlöder and D'Croz 2004 [8]	30 days	1 °C	*Pocillopora damicornis*	Protein	n.s.
			Porites lobata	Protein	n.s.
Gibbin et al. 2018 [9]	7 days	2.5 °C	*Pocillopora damicornis*	P_{gross}	n.s.
				R	n.s.
				Protein	n.s.
				δ13C	Warm-acclimated: increase in coral gastrodermis (14 %)
				δ15N	Warm-acclimated: decrease in coral gastrodermis (- 81 %)

Grover et al. 2011 [10]	10 days	3 °C and 7°C	*Stylophora pistillata*	Protein	3/7 °C: n.s.
				P_{gross}	3 °C: n.s.<br>7 °C: -200 %
				R	3/7 °C: n.s.
Rodrigues and Grottoli 2006 [11]	1 month	3 °C	*Montipora capitata*	δ13C (host)	n.s.
				δ13C (symbiont)	decrease (- 2 ‰)
				δ15N	n.s.
			Porites compressa	δ13C	n.s.
				δ15N	n.s.
Rodrigues and Grottoli 2007 [12]	1 month	3 °C	*Montipora capitata*	Protein	n.s.
				P_{gross}	Decrease (- 67 %)
				R	Decrease (- 45 %)
			Porites compressa	Protein	Decrease (- 36 %)
				P_{gross}	n.s.
				R	n.s.
Nyström et al. 2001 [13]	24 h	4 °C	*Porites cylindrica*	P_{gross}	Decrease (- 50 %)
				R	Decrease (- 57 %)
Bahr et al. 2018 [14]	1 day	4 °C	*Montipora capitata*	P_{net}	n.s.
				R	n.s.
				P/R	Decresase (- 23 %)
			Pocillopora damicornis	P_{net}	n.s.
				R	Increase (+ 40 %)
				P/R	Decrease (- 32 %)
			Leptastrea purpurea	P_{net}	Decrease (- 27 %)
				R	n.s.
				P/R	Decrease (- 39 %)
Hoadley et al. 2015 [15]	24 days	5 °C	*Acropora millepora*	Protein	Decrease (- 31 %)
				Fv/Fm	Decrease (- 14 %)
				P/R	n.s.
				R	n.s.
			Pocillopora damicornis	Protein	n.s.
				Fv/Fm	Decrease (- 10 %)
				P/R	n.s.
				R	Increased (+ 47 %)
			Montipora monasteriata	Protein	Decrease (- 28 %)
				Fv/Fm	Decrease (- 15 %)
				P/R	n.s.
				R	Increased (+ 62 %)
			Turbinaria reniformis	Protein	Increase (+ 83 %)
				Fv/Fm	n.s.
				P/R	n.s.
				R	Increased (+ 31 %)
Petrou et al. 2018 [16]	12 days	5 °C	*Acropora millepora*	Fv/Fm	Significant decrease from day 9 onwards (- 35 %)
Béraud et al. 2013 [17]	7 days	5 °C	*Turbinaria reniformis*	Protein	n.s.
				P_{gross}	- 100 %
				R	+ 64 %
				δ13C	n.s.
				δ15N	n.s.
Courtial et al. 2017 [18]	5 weeks	5 °C	*Pocillopora damicornis*	Protein	Decrease (- 60 %)
				P_{net}	n.s.
				R	n.s.
			Turbinaria reniformis	Protein	n.s.
				P_{net}	n.s.
				R	n.s.

Ferrier-Pagès et al. 2010 [19]	5 days	5 °C	*Stylophora pistillata*	Fv/Fm	Decrease (- 18 %)
				P_{gross}	Decrease (- 60 %)
				R	Decrease (- 33 %)
			Turbinaria reniformis	Fv/Fm	Decrease (- 11 %)
				P_{gross}	Decrease (- 75 %)
				R	Decrease (- 66 %)
			Galaxea fascicularis	Fv/Fm	Decrease (- 33 %)
				P_{gross}	Decrease (- 51 %)
				R	Decrease (- 33 %)
Baker et al. 2018 [20]	10 days	5 °C	*Orbicella faveolata (shallow samples)*	% C content	Host: Decrease (- 15 %) Symbiont: n.s.
				δ15N (host)	n.s.
				δ15N (symbiont)	Increase (+ 32 %)
				δ13C (host)	n.s.
				δ13C (symbiont)	Increase (+ 14 %)
Hoogenboom et al. 2012 [21]	10-13 days	6 °C	*Stylophora pistillata*	P_{gross} (unfed)	Decrease (- 47 %)
				P_{gross} (fed)	n.s.
				R	n.s.
			Turbinaria reniformis	P_{gross} (unfed)	Decrease (- 39 %)
				P_{gross} (fed)	n.s.
				R	n.s.
Grottoli et al. 2017 [22]	37 days	6 °C	*Stylophora pistillata*	Protein	n.s.
				δ13C (host)	Increase (+ 2 ‰)
			Pocillopora damicornis	Protein	n.s.
				δ13C (host)	n.s.
			Favia favus	Protein	Decrease (- 48 %)
				δ13C (host)	Increase (+ 2 ‰)
COMBINED EXPERIMENTS					
Hall et al. 2018 [23]	30 days	5 °C Nutrients: nitrate+nitrite (1µM)+PO_4 (0.0625 µM)	*Stylophora pistillata*	Fv/Fm	Temp.: decrease (NA) Nutrients: n.s. Comb.: n.s.
				P_{net}	Temp.: n.s. Nutrients: decrease (- 34 %) Comb.: n.s.
				R	n.s.
				Protein	Temp.: n.s. Nutrients: decrease (- 24 %) Comb.: n.s.
Ezzat et al. 2016 [24]	4 weeks eutrophication, 10 days warming	DIP: 2µM Temp.: 5 °C	*Pocillopora damicornis*	R	DIP: - 50 % Temp.: + 25 % Comb.: + 44 %
				P_{gross}	DIP: n.s. Temp.: - 50 % Comb.: - 50 %
Present study	5 weeks, Temp.: 3 weeks	PO_4: 1,2,8 µM Temp.: 6 °C	*Xenia umbellata*	P_{gross}	n.s.
				R	n.s.
				P/R	n.s.
				Protein	Temp.: decrease (- 62 %)
				δ13C	Temp.: Decrease (- 6.9 %)
				δ15N	Temp.: Decrease (- 10 %)

References

1. Ferrier-Pagès, C., Gattuso, J. P., Dallot, S. & Jaubert, J. Effect of nutrient enrichment on growth and photosynthesis of the zooxanthellate coral Stylophora pistillata. *Coral Reefs* **19**, 103–113 (2000).

2. Koop, K. *et al.* ENCORE: The Effect of Nutrient Enrichment on Coral Reefs. Synthesis of Results and Conclusions. *Mar. Pollut. Bull.* **42**, 91–120 (2001).

3. Stambler, N., Popper, N., Dubinsky, Z. & Stimson, J. Effects of nutrient enrichment and water motion on the coral Pocillopora damicornis. *Pacific Sci.* **45**, 299–307 (1991).

4. Tanaka, Y., Grottoli, A. G., Matsui, Y., Suzuki, A. & Sakai, K. Effects of nitrate and phosphate availability on the tissues and carbonate skeleton of scleractinian corals. *Mar. Ecol. Prog. Ser.* **570**, 101–112 (2017).

5. Hoegh-Guldberg, O., Muscatine, L., Goiran, C., Siggaard, D. & Marion, G. Nutrient-induced perturbations to δ13C and δ15N in symbiotic dinoflagellates and their coral hosts. *Mar. Ecol. Prog. Ser.* **280**, 105–114 (2004).

6. Silbiger, N. J. *et al.* Nutrient pollution disrupts key ecosystem functions on coral reefs. *Proc. R. Soc. B Biol. Sci.* **285**, (2018).

7. Krueger, T. *et al.* Common reef-building coral in the Northern Red Sea resistant to elevated temperature and acidification. *R. Soc. Open Sci.* **4**, (2017).

8. Schlöder, C. & D'Croz, L. Responses of massive and branching coral species to the combined effects of water temperature and nitrate enrichment. *J. Exp. Mar. Bio. Ecol.* **313**, 255–268 (2004).

9. Gibbin, E. M. *et al.* Short-Term Thermal Acclimation Modifies the Metabolic Condition of the Coral Holobiont. *Front. Mar. Sci.* **5**, (2018).

10. Grover, R. *et al.* Coral uptake of inorganic phosphorus and nitrogen negatively affected by simultaneous changes in temperature and pH. *PLoS One* **6**, 1–10 (2011).

11. Rodrigues, L. J. & Grottoli, A. G. Calcification rate and the stable carbon, oxygen, and nitrogen isotopes in the skeleton, host tissue, and zooxanthellae of bleached and recovering Hawaiian corals. *Geochim. Cosmochim. Acta* **70**, 2781–2789 (2006).

12. Rodrigues, L. J. & Grottoli, A. G. Energy reserves and metabolism as indicators of coral recovery from bleaching. *Limnol. Oceanogr.* **52**, 1874–1882 (2007).

13. Nyström, M., Nordemar, I. & Tedengren, M. Simultaneous and sequential stress from increased temperature and copper on the metabolism of the hermatypic coral Porites cylindrica. *Mar. Biol.* **138**, 1225–1231 (2001).

14. Bahr, K. D., Rodgers, K. S. & Jokiel, P. L. Ocean warming drives decline in coral metabolism while acidification highlights species-specific responses. *Mar. Biol. Res.* **14**, 924–935 (2018).

15. Hoadley, K. D. *et al.* Physiological response to elevated temperature and pCO2 varies across four Pacific coral species: Understanding the unique host+symbiont response. *Nat. Publ. Gr.* **5**, 1–15 (2015).

16. Petrou, K., Nielsen, D. A. & Heraud, P. Single-Cell Biomolecular Analysis of Coral Algal Symbionts Reveals Opposing Metabolic Responses to Heat Stress and Expulsion. *Front. Mar. Sci.* **0**, 110 (2018).

17. Béraud, E., Gevaert, F., Rottier, C. & Ferrier-Pagès, C. The response of the scleractinian coral Turbinaria reniformis to thermal stress depends on the nitrogen status of the coral holobiont. *J. Exp. Biol.* **216**, 2665–2674 (2013).

18. Courtial, L., Roberty, S., Shick, J. M., Houlbrèque, F. & Ferrier-Pagès, C. Interactive effects of ultraviolet radiation and thermal stress on two reef-building corals. *Limnol. Oceanogr.* **62**, 1000–1013 (2017).

19. Ferrier-Pagès, C., Rottier, C., Beraud, E. & Levy, O. Experimental assessment of the feeding effort of three scleractinian coral species during a thermal stress: Effect on the rates of photosynthesis. *J. Exp. Mar. Bio. Ecol.* **390**, 118–124 (2010).

20. Baker, D. M., Freeman, C. J., Wong, J. C. Y., Fogel, M. L. & Knowlton, N. Climate change promotes

parasitism in a coral symbiosis. *ISME J.* **12**, 921–930 (2018).

21. Hoogenboom, M. O., Campbell, D. A., Beraud, E., DeZeeuw, K. & Ferrier-Pagès, C. Effects of light, food availability and temperature stress on the function of photosystem II and photosystem I of coral symbionts. *PLoS One* **7**, (2012).

22. Grottoli, A. G., Tchernov, D. & Winters, G. Physiological and Biogeochemical Responses of Super-Corals to Thermal Stress from the Northern Gulf of Aqaba, Red Sea. *Front. Mar. Sci.* **4**, (2017).

23. Hall, E. R. *et al.* Eutrophication may compromise the resilience of the Red Sea coral Stylophora pistillata to global change. *Mar. Pollut. Bull.* **131**, 701–711 (2018).

24. Ezzat, L., Maguer, J.-F. F., Grover, R. & Ferrier-Pagès, C. Limited phosphorus availability is the Achilles heel of tropical reef corals in a warming ocean. *Sci. Rep.* **6**, 1–11 (2016).
